# Ramsay Hunt syndrome following otoplasty

**DOI:** 10.1590/S1808-86942011000600022

**Published:** 2015-10-19

**Authors:** Marco Antônio Rios Lima, Jacinto de Negreiros Júnior

**Affiliations:** 1 3rd-Year ENT Resident (R3); 2 MSc. Assistant Physician and Coordinator of the Medical Residency Program in Otorhinolaryngology of the Armed Forces Hospital in Brasília. Armed Forces Hospital in Brasília

**Keywords:** ear, external, herpes zoster, herpes zoster oticus

## INTRODUCTION

The Ramsay-Hunt syndrome (RHS) is characterized by the association of herpes-zoster oticus with acute peripheral facial paralysis^1^. Its physiopathology is associated with the reactivation of the varicella-zoster virus (VZV) in the geniculate ganglion of the facial nerve^1^,^2^.

Different surgical procedures have been described in the literature as triggers for virus reactivation^1^,^2^.

Below, we describe what we believe to be the first case reported of RHS happening after otoplasty, focusing on its diagnosis, treatment and evolution.

## CASE PRESENTATION

A twelve year old boy, previously healthy, was submitted to bilateral otoplasty in order to correct protruding ears. There were no initial complications and the suture was removed 14 days after the procedure (PO).

**Figure 1:**
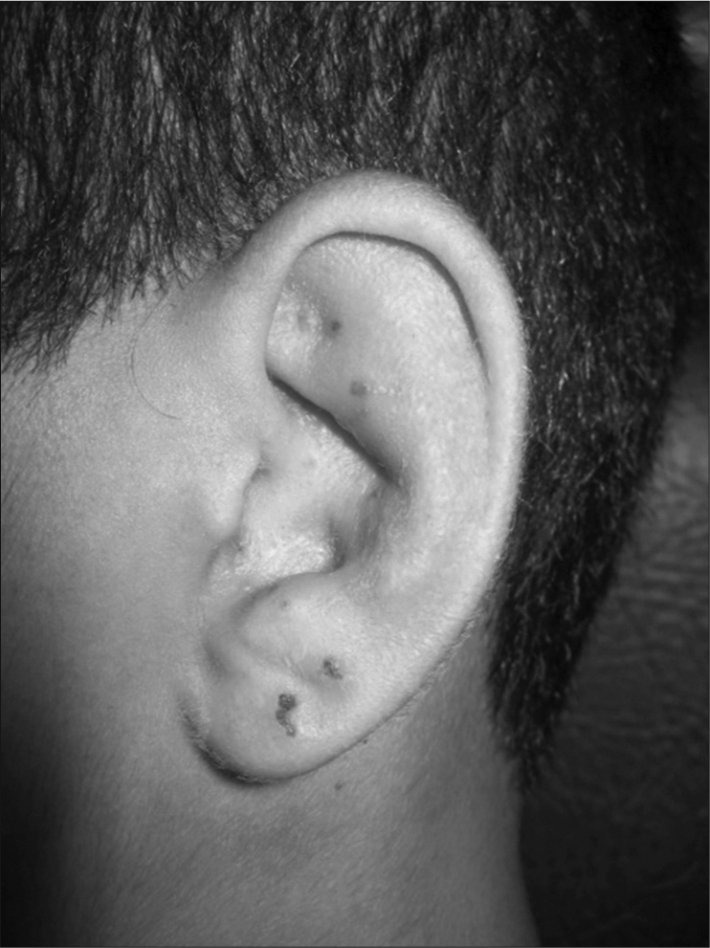
Herpes-zoster oticus.

On the 25th day of post-op, he started having a little difficulty in shutting his left eye. The patient reported having noticed vesicles in his left ear pinna, which had been there for three days, together with ear pain and ipsilateral hearing loss. Two days later, he reported difficulties in moving his face on the left side. He had had varicella infection at 4 years of age, without complications. Upon physical exam, he had House Brackmann level III left-side facial palsy and crusty lesions on his left ear pinna (Figure 1). Upon otoscopy, we noticed diffuse edema and hyperemia in his left external ear canal, making it impossible to see the tympanic membrane. His right ear pinna and ipsilateral otoscopy were normal. His mouth and anterior rhinoscopy did not show changes. He had a negative Rinne test on the left side with the Weber test lateralized to the same side.

We started treating his RHS with Dexamethasone 4mg/day per os, during 7 days, with progressive weaning (28 days of steroid treatment according to our protocol), Acyclovir 2g/day (7 days) and eye lubrication drops on the left side. The external otitis was treated with ear drops (Ciprofloxacin 0.2% + hydrocortisone 1% - 10 days). We ordered a CBC and serology tests. One week later, he had complete symptom resolution in his left ear pinna as well as his ipsilateral external otitis. Tonal audiometry showed normal hearing thresholds in both ears. CBC showed leukocytosis (16,100) without deviations.

Serology tests were positive for IgG and negative for IgM for Herpes I and II, mononucleosis and cytomegalovirus; negative serology for HIV. He progressively recovered facial movement and had complete palsy resolution upon the fourth week after the diagnosis. Herpes-zoster serology (enzyme immune assay) was carried out only one month after symptom onset, which revealed negative IgM and positive IgG in high titers (2.556 – reference value is up to 0.2).

## DISCUSSION

The RHS is rare in childhood, with an incidence of approximately 5 cases/100,000 inhabitants/year. Nonetheless, it is the second most common cause of non-traumatic facial paralysis.^3^

Today it is believed that virus reactivation is an important cause of facial paralysis in late post-op^1^. After the prime-infection, the VZV remains latent in the sensorial ganglion of the facial (VII) and trigeminal (V) nevers^2^. Surgical procedures in the vicinities of the facial nerve, such as in the middle and inner ears, trigger viral reactivation and facial paralysis because of the direct stimulation of the VII cranial nerve^1^. There are also cases of late peripheral facial paralysis induced by the VZV after orofacial surgeries and dental procedures, and in these cases the hypothesis is for an ascending viral infection through the lingual nerve^2^.

We may hypothesize that the otoplasty procedure triggered the viral reactivation through direct stimulation of the VII cranial nerve (ear pinna)^4^, or through the stimulation of the V nerve (auricle-temporal branch)^4^, which would transmit the stimulus through the chorda tympani nerve all the way to the facial nerve.

Diagnosis is fundamentally clinical^5^. Complementary tests may corroborate or confirm the diagnosis. In the present case, serologic diagnosis could not be established because of the difficulty in performing serology at the time of diagnosis. Nonetheless, high titers of anti-VZV IgG antibodies found one month after symptom onset are highly suggestive of virus reactivation. Serologic response is not always detected in RHS patients.^2^ There are reports of variable patterns of immune response from the virus host^6^.

## FINAL REMARKS

We described here a case of a patient who developed RHS in the late postoperative of bilateral otoplasty, having complete symptom remission after clinical treatment. The close follow up of patients submitted to otorhinolaryngological and neck/facial surgeries is highly important in order to make an early diagnosis and to provide proper treatment aiming at optimizing prognosis.
